# Pneumocephalus Secondary to Sternutation: A Case Report

**DOI:** 10.5811/cpcem.48669

**Published:** 2026-02-25

**Authors:** Tushar Tejpal, John Ashurst, Danielle Barnett-Trapp

**Affiliations:** * Arizona College of Osteopathic Medicine, Midwestern University, Glendale, Arizona; † Kingman Regional Medical Center, Department of Emergency Medicine, Kingman, Arizona

**Keywords:** sternutation, pneumocephalus, cribriform meningoencephalocele, cerebrospinal fluid leak, case report

## Abstract

**Introduction:**

Sternutation is a physiological reflex that clears the upper respiratory tract through forceful air expulsion. Although it is typically considered benign, sternutation can generate substantial pressure and airflow that can result in barotrauma, including pneumocephalus.

**Case Report:**

A 67-year-old female presented with shortness of breath, rhinorrhea, and a headache following sneezing. Physical exam revealed no signs of trauma or neurological deficits but did note clear rhinorrhea bilaterally. Computed tomography (CT) of the head revealed extensive extra-axial intracranial gas bilaterally, and the patient was admitted for further management. While admitted, otolaryngology was consulted and surgically corrected a right cribriform meningoencephalocele with an active cerebrospinal fluid leak. At follow-up the patient had no residual rhinorrhea symptoms or focal neurological findings.

**Conclusion:**

One proposed mechanism of sternutation-induced pneumocephalus involves the “one-way-ball-valve” effect, whereby elevated sinus pressure during sternutation forces air through a dural defect, trapping it within the cranial cavity. Diagnosis is typically made with non-contrast CT and treatment depends on severity, ranging from conservative oxygen therapy to urgent surgical intervention. Indications on CT, such as the Mount Fuji sign, air bubble sign, and the peaking sign, help differentiate tension pneumocephalus from less severe forms. This case adds to the growing literature on sternutation-induced pneumocephalus and highlights the importance of recognizing sternutation as a potential source for serious intracranial pathology.

## INTRODUCTION

Although rare, sternutation has been identified as a potential trigger for spontaneous pneumocephalus.^1^–^3^ Sternutation is a reflex mechanism designed to clear irritants from the upper respiratory tract, particularly the nasal cavity.^4^ The process begins with sensory input from nasal mucosa receptors, which activate branches of the trigeminal nerve.^4^ These signals are relayed to the sneezing center in the brainstem, which then coordinates the efferent response leading to contraction of respiratory muscles and forceful air expulsion.^4^

Sternutation generates substantial pressure and airflow.^3^ A typical sneeze produces an intranasal pressure around 1 kilopascal (kPA) with air expelled up to 30 meters per second.^3^ However, suppressing a sneeze can increase pressures dramatically, up to 21.8 kPA.^3^ This force may lead to barotrauma, including orbital fractures, pneumothorax, subcutaneous emphysema and, in rare cases, pneumocephalus.^3^ We describe a case of pneumocephalus occurring after forceful sneezing, illustrating how a benign reflex can result in serious intracranial complications.

## CASE REPORT

A 67-year-old female with a past medical history of diabetes, hypertension, and hyperlipidemia presented to the office of a family medicine physician with a chief complaint of a two-week cough with shortness of breath. While in the office, she was found to have a pulse oxygenation of 88% on room air and was subsequently transferred to the emergency department (ED). Upon presentation to the ED, she also complained of subjective fevers, rhinorrhea, and a headache that occurred after an episode of sneezing. She denied any recent trauma or any other neurological symptoms.

Upon examination, her vital signs were as follows: temperature, 99.3 °Fahrenheit; heart rate, 74 beats per minute; respiratory rate, 16 breaths per minute; blood pressure, 166/71 millimeters of mercury; and pulse oxygenation, 91% on room air. No outward signs of trauma were visible, and she was alert and oriented. Her mucous membranes were moist with clear rhinorrhea bilaterally, and no hemotympanum was appreciated. Her cranial nerves were equal and symmetrical bilaterally, and she had 5/5 muscle strength in the upper and lower extremities. While in the ED, she was able to ambulate without signs of ataxia.

**Image 1:**
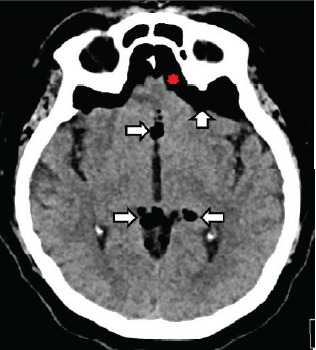
Axial image of non-contrast computed tomography of the head depicting intracranial air (white arrows). The red asterisk indicates the Mount Fiji sign, which is defined by frontal lobe separation due to subdural air.

**Image 2:**
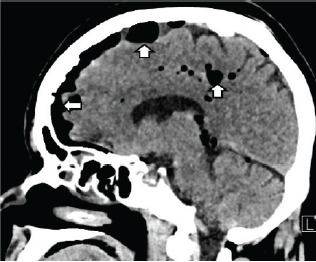
Sagittal image from the patient’s non-contrast computed tomography of the head depicting intracranial air (white arrows).

Initial laboratory studies revealed an elevated white blood cell count, 20.4 × one billion particles per liter (10^9^/L) (reference range: 4.5–11.0 × 10^9^/L) with associated hyponatremia, 129 millimoles per liter (mmol/L) [136–146 mmol/L]), and hypochloremia 90 mmol/L (95–105 mmol/L). Chest radiography showed bilateral perihilar and left basilar infiltrates indicative of pneumonia. Computed tomography (CT) of the head showed a moderate to large amount of extra-axial intracranial gas bilaterally in multiple sulci and the basal cistern with the left greater than the right (Images 1 and 2).

After discussion with neurosurgery, the patient was given two grams of cefepime and one gram of vancomycin, and she was admitted for further evaluation and management. During her hospitalization, otolaryngology was consulted and surgically corrected a right cribriform meningoencephalocele with an active cerebrospinal fluid (CSF) leak that measured < 5 mm. She was discharged from the hospital after 20 days and at primary care follow-up had no residual neurological findings.

## DISCUSSION

While the reflex mechanics of sternutation are well understood, its potential to cause serious injury is often overlooked.^3^ Case reports increasingly describe sternutation-related injuries, including rare complications such as pneumocephalus and intracranial hemorrhage.^1^–^3^ Notably, these injuries have been reported even in patients with no underlying comorbidities, emphasizing that forceful sternutation can result in significant trauma in healthy individuals.^3^ One proposed mechanism is the “one-way ball-valve” theory, which suggests that elevated pressure in the sinuses during sternutation can force air through a dural defect, trapping air within the cranial cavity.^1^ This Valsalva-like effect may redirect pressure toward vulnerable areas and can result in barotrauma.^1^,^3^

*CPC-EM Capsule*

What do we already know about this clinical entity?

*Sternutation is a well described reflex however, it rarely triggers intracranial*

*complications such as pneumocephalus*.

What makes this presentation of disease reportable?

*We report a rare case of pneumocephalus triggered by sternutation with underlying*

*cribriform meningoencephalocele and active cerebrospinal fluid leak which was surgically repaired*.

What is the major learning point?

*Even as a benign reflex, sternutation can cause life threatening pneumocephalus in*

*patients with skull base defects and therefore requires early recognition*.

How might this improve emergency medicine practice?

*It promotes awareness of sternutation-induced pneumocephalus, guiding emergency medicine physicians to consider nontraumatic causes when intracranial air is detected*.

Non-contrast CT of the head, the gold standard for diagnosis of pneumocephalus, is capable of detecting as little as 0.55 mL of air due to the low Hounsfield unit (HU) of air (−1000 HU).^1^ There are certain CT findings that help to differentiate mild cases from tension pneumocephalus.^1^,^6^ The Mount Fuji sign is characterized by separation of the frontal lobes due to subdural air and is highly specific for tension pneumocephalus.^1^,^6^ Additionally, the air bubble sign (when air is seen within the basal cistern) suggests extensive air dispersion.^1^,^6^ In contrast, the peaking sign shows frontal lobe compression without separation and indicates a less critical state.^1^,^6^ Although magnetic resonance imaging can be used in some cases, it is generally less sensitive for detecting air.^1^ Similarly, skull radiographs occasionally reveal pneumocephalus but are limited in sensitivity and often miss smaller volumes of intracranial air.^1^

Pneumocephalus in the context of a cribriform meningoencephalocele with active CSF leakage highlights the serious risks associated with defects to the skull base.^7^ Cerebrospinal fluid leakage creates a negative intracranial pressure that predisposes patients to pneumocephalus, while also increasing the risk of infections such as meningitis.^7^,^8^ In our patient, a sudden increase in intranasal pressure during sternutation may have allowed air to enter the intracranial space through a structurally weakened cribriform plate, resulting in pneumocephalus. This finding underscored the importance of preoperative imaging to evaluate the location and extent of the skull base defect and guide surgical planning, which ultimately led to successful repair and prevention of further complications.

The treatment of spontaneous pneumocephalus depends on the severity of symptoms.^1^ While many cases can be managed conservatively with observation and oxygen therapy, surgical intervention is indicated for symptomatic, recurrent, or tension pneumocephalus.^1^ Tension pneumocephalus is a neurosurgical emergency requiring immediate decompression due to the risk of brain herniation.^1^,^9^ Surgical options include burr hole trephination, needle aspiration and craniotomy.^1^,^9^ The primary goal of surgical intervention is to evacuate the air and repair any underlying defects in the skull base or dura.^1^ Intraoperative techniques may include endoscopic or open approaches depending on the defect location and surgeon expertise.^9^ Patient education is also essential. Avoiding activities that increase intracranial pressure, such as nose blowing or the Valsalva maneuver, helps reduce the risk of recurrence.^1^

Emerging trends in the management of pneumocephalus include both non-invasive and surgical advancements. High-flow nasal cannula oxygen therapy has shown promise as a more effective and comfortable alternative to traditional oxygen delivery methods.^10^–^11^ High-flow nasal cannula delivers heated, humidified oxygen that reduces nasal irritation, improves patient comfort, and provides consistently high flow rates that help to maintain stable oxygen and fraction of inspired oxygen.^10^ This high oxygen delivery also creates a strong gradient for nitrogen to be washed out, helping to remove trapped intracranial air more efficiently.^10^ For surgical management, endoscopic endonasal approaches are increasingly being used for skull base repair, particularly for areas such as the cribriform plate that are difficult to access.^9^ Endoscopic assistance helps improve visualization and allows for more precise repairs, potentially reducing the risk of CSF leak.^9^

## CONCLUSION

This case highlights a rare occurrence of non-traumatic pneumocephalus secondary to forceful sternutation. Although sternutation is a physiologic reflex, in rare instances it can lead to serious complications such as pneumocephalus. Emergency clinicians should consider this diagnosis in patients presenting with acute neurological symptoms following barotrauma-like events. Early recognition, timely imaging, and individualized management (ie, conservative or surgical), are essential to achieving optimal outcomes in these uncommon but serious cases.
